# Disparities in use of physical restraint and chemical sedation in the emergency department by patient housing status

**DOI:** 10.1371/journal.pone.0319286

**Published:** 2025-03-13

**Authors:** Leah Robinson, Caitlin R. Ryus, Bidisha Nath, Anusha Kumar, Riddhi Desai, Dhruvil Shah, Isaac V. Faustino, Ambrose H. Wong

**Affiliations:** 1 Department of Emergency Medicine, Yale School of Medicine, New Haven, Connecticut, United States of America; 2 Department of Health Policy and Management, The Johns Hopkins Bloomberg School of Public Health, Baltimore, Maryland, United States of America; Wingate University, UNITED STATES OF AMERICA

## Abstract

**Background:**

A growing body of research has found there to be disproportionate physical restraint and chemical sedation use for historically marginalized populations in the emergency department (ED). This association has been examined with regard to patient race, ethnicity, sex, and age. Preliminary research has highlighted the ways in which unhoused status may also relate to the use of physical restraint and chemical sedation in the ED. Given the adverse health outcomes associated with these methods in the ED, further research is needed to explore the relationship between patient housing status and physical restraint/chemical sedation use in more depth.

**Methods:**

We conducted a cross-sectional study of all ED visits among patients aged 18 years of age and older presenting to eight hospitals within a regional healthcare network in New England between January 1, 2013, and December 31, 2021. Descriptive statistics and mixed effects logistic regression models nesting by patient were used to characterize the relationship between housing status and likelihood of restraint and/or sedation use.

**Findings:**

Restraint orders were found in 3,160 (5.7%) visits by unhoused patients, compared to 44,155 (1.5%) for housed patients. Unhoused status was significantly associated with restraint/sedation use (adjusted odds ratio =  1.45, 95% CI 1.36-1.54).

**Conclusion:**

Our study identified a significant association between housing status and ED restraint and sedation use after adjusting for demographic factors and chief complaints. This finding has important implications pertaining to the care of unhoused patients in the ED and for examination of structural factors like housing status and their impact on psychiatric emergency care.

## Introduction

Physical restraints and chemical sedatives are often used in the emergency department (ED) to prevent injury and physical danger for patients in behavioral crises or with symptoms of agitation, defined as excessive psychomotor activity leading to aggressive or violent behavior [1]. While restraints and sedation may be required to prevent harm, their use is associated with adverse health outcomes, such as physical injury, cardiac arrest, and lasting psychological trauma and distress [2]. A growing body of research also indicates that there is disproportionate use of physical restraint and chemical sedation for historically marginalized populations in the ED. Specifically, elevated restraint odds are associated with Black race, male sex, and lower socioeconomic status, likely due to systemic, structural, and implicit biases [3].

Less is known about how social factors, such as housing status, influence levels of restraint and sedation use. A small number of studies have included housing status as a variable in models examining associations with restraint use, yielding mixed results. For example, studies by Carreras Tartak et. al (2021) and Pino et al. (2024) did not find a significant association between homelessness and physical restraint after adjusting for covariates [4,5]. Yet, a study by Schnitzer et al. (2020) found that people experiencing homelessness (PEH) were nearly six times more likely to be restrained than those who were stably housed (RR =  5.77, 95% CI =  5.06 to 6.58), and a study by Stillman et al. (2023) found odds of restraint to be 1.7 times higher (95% CI =  1.44 to 2.00) for PEH compared to those who were stably housed [6,7]. The small sample sizes of PEH included these studies may partly explain the discrepancies in findings. Additionally, differences in clinical settings (e.g., academic vs. safety-net hospitals) may also contribute, as research has found PEH may receive better treatment at safety-net or other hospitals serving a higher proportion of unhoused patients [8].

Our prior qualitative work among patients who were restrained has highlighted unhoused status as particularly linked to the use of restraint and sedation in the ED [9]. PEH have cited their housing status as contributing to stigmatization, discrimination, and dehumanization in their encounters with medical personnel [10]. Furthermore, restraint use often occurs in the setting of chemical intoxication and acute exacerbations of mental illness, emergency conditions that are overrepresented among PEH in the ED [11]. While our team did include housing status as a covariate in a study conducted in 2021 examining the association between race/ethnicity and restraint use, housing status was not the central focus of this prior study [3]. Moreover, since our 2021 study, our approach to identifying homelessness has been significantly refined to now use a comprehensive algorithm incorporating ICD-10 codes, chief complaints, and address data, addressing known limitations of prior research. We have also significantly expanded the timeframe and number of hospitals included in our dataset to now include data from eight hospitals and over 55,000 ED visits from unhoused patients, compared to three hospitals and 2,970 visits from unhoused patients included in our prior study. The expansion of our dataset increases the generalizability of our findings and provides sufficient statistical power to explore nuanced relationships and examine housing status as a key factor in restraint use. This allows the present analysis to add significant value beyond what was offered by our prior study.

In this study, we therefore specifically examined the association between housing status (unhoused vs. housed) and restraint and sedation use in the ED. We hypothesized that unhoused patients would have higher odds of being restrained and/or sedated than housed patients. By leveraging a large and diverse study population, this study makes a meaningful contribution to the literature on disparities in ED care for patients who are unhoused and deepens our understanding of a relationship where existing research findings are currently sparce and mixed.

## Methods

### Study design and population

We conducted a cross-sectional study of all ED visits among patients aged 18 years of age and older presenting to eight hospitals within a regional healthcare network in New England between January 1, 2013, and December 31, 2021. The regional healthcare network included two nonacademic urban, two academic urban, and four nonacademic suburban sites. All data were electronically abstracted directly from patients’ EPIC Systems (Verona, WI, USA) electronic health record (EHR). The primary exposure of interest was housing status and the primary outcome of interest was violent physical restraint or chemical sedation use.

### Main outcomes and measures

The primary outcome of interest in this study was a dichotomous variable representing the presence of a violent restraint order and/or a completed order for an intramuscular (IM) sedative in a patient’s EHR record during an ED visit. Our study broadly defines restraint use to include either the presence of a violent restraint or chemical sedation order to capture all instances of agitation being treated through methods other than verbal de-escalation. Violent physical restraints were indicated for management of behavior that jeopardized the immediate physical safety of the patient, staff, or others [12]. Chemical sedation was defined as the administration of IM antipsychotics (haloperidol, droperidol, olanzapine), benzodiazepines (lorazepam, midazolam), ketamine, and/or diphenhydramine (in conjunction with an antipsychotic), as previously detailed in our prior work [13]. Chemical sedation did not indicate use for chemical restraint or for restriction of patient movement. Non-violent restraint orders, which are often used to promote medical healing or for the protection of equipment, were not included in our primary outcome [3]. We also examined violent restraint and chemical sedation as separate primary outcomes in additional sub-analyses (Tables in S1 Table and S2 Table).

The primary exposure of interest in this study was the binary variable of housing status (housed vs. unhoused). Patients were identified as unhoused within our EHR based on a rigorous query using a combination of ICD-10 codes for homelessness in the clinical diagnosis, chief complaint of homelessness, and address criteria including having no address, using the hospital address, or an address corresponding to a local shelter [14]. This aligned with the criteria employed by our hospital’s dedicated homelessness social work team, which have demonstrated operational accuracy by increasing the administrative identification of homelessness within the hospital system by 50%. Additional variables in our model were defined a priori and included sex, age, race and ethnicity, and primary chief complaint. These covariates were selected because they are the primary variables that may confound the relationship between housing status and restraint/sedation use. Chief complaints were manually grouped into six categories (i.e., medical/non-behavioral, trauma, cognitive or neurological, alcohol/drug use, psychiatric, and agitation) in accordance with prior work regarding agitation and restraint use in the ED [15].

### Statistical analysis

Descriptive statistics were used to summarize patient demographics, clinical characteristics, housing status, and frequency of restraint and sedation use. Univariate and multivariable mixed-effects logistic regression models were then used to examine the association between the outcome (restraint or sedation used =  1, no restraint or sedation used =  0) and the main exposure of interest (unhoused =  1, housed =  0). Univariate mixed-effects logistic regression models were used to explore the relationship between restraint and sedation use and the main exposure of housing status, as well as with all other suspected confounders (i.e., age, sex, race and ethnicity, and primary chief complaint).

A mixed-effects logistic regression model, clustered by patient, was selected to account for complexities of evaluating ED restraint use among a patient population with a high prevalence of risk factors for restraints. This model-type centers on patient characteristics and accounts for repeated visits by clustering by individual patients. This method mitigates overfitting while providing a robust framework to examine the primary association of interest relative to the probability of being restrained based on patient level characteristics. Analyses were conducted using STATA 18.0 software. Data used in the study were accessed on March 5, 2024. Authors had access to participants’ identifying information. The study was approved by our human investigation committee and followed the STROBE reporting guideline.

## Results

A total of 2,977,672 unique ED visits among 837,119 patients were included in our study. Among all visits, 55,350 (1.9%) were by patients who were unhoused (Table 1). Unhoused patients were more likely to be male (72.2% vs. 44.5%), Black (34.0% vs. 22.2%), and have chief complaints related to substance use (26.7% vs. 3.7%) or psychiatric conditions (21.6% vs. 4.4%). Violent restraint and/or chemical sedation orders were found in 47,315 (1.6%) of visits by all patients. Among visits by unhoused patients, 3,160 (5.7%) maintained a restraint and/or sedation order. Among visits by patients who were stably housed, 44,155 (1.5%) of visits maintained a restraint and/or sedation order. After nesting by patient and adjusting for age, race and ethnicity, sex, and chief complaint, unhoused status was found to be significantly associated with restraint/sedation use (Table 2) (adjusted odds ratio =  1.45, 95% CI 1.36-1.54). The association between unhoused status and restraint and/or sedation remained significant when examining violent restraint and sedation separately as primary outcomes (Tables in S1 Table and S2 Table).

**Table 1:** Demographic and visit characteristics of emergency department patients by housing status, January 2013 - December 2021.

	Housing Status No. (%) N = 2,977,672	
	**Housed** N = 2,922,322 (98.1)	**Unhoused** N = 55,350 (1.9)
**Age**		
18-25	407,864 (14.0)	4,170 (7.5)
26-35	497,736 (17.0)	11,968 (21.6)
36-45	418,877 (14.3)	13,667 (24.7)
46-55	465,102 (15.9)	14,840 (26.8)
56-64	384,517 (13.2)	7,959 (14.4)
65+	748,226 (25.6)	2,746 (5.0)
**Sex**		
Female	1,623,225 (55.5)	15,393 (27.8)
Male	1,299,097 (44.5)	39,957 (72.2)
**Race Ethnicity**		
White Non-Hispanic	1,542,127 (52.8)	22,737 (41.1)
AI/AN Non-Hispanic	8,120 (0.3)	202 (0.4)
Asian Non-Hispanic	47,378 (1.6)	163 (0.3)
Black Non-Hispanic	647,574 (22.2)	18,820 (34.0)
Hispanic or Latina/o/x	596,126 (20.4)	11,956 (21.6)
Missing or Unknown	13,214 (0.5)	718 (1.3)
Native Hawaiian/PI Non-Hispanic	2,834 (0.1)	44 (0.1)
Other Non-Hispanic	64,949 (2.2)	710 (1.3)
**Chief Complaint**		
Medical/Non-Behavioral	2,348,162 (80.4)	30,940 (56.0)
Trauma	441,554 (15.1)	5,245 (9.5)
Cognitive or Neurologic	200,726 (6.9)	3,682 (6.7)
Alcohol/Drug Use	107,627 (3.7)	14,771 (26.7)
Psychiatric	127,178 (4.4)	12,053 (21.6)
Agitation	10,367 (0.4)	903 (1.6)
**Presence of Restraint and/or Sedation**		
	44,155 (1.5)	3,160 (5.7)

**Table 2:** Descriptive, univariate and adjusted multivariable mixed effects logistic regression models of physical restraint and/or chemical sedation order in the emergency department, January 2013 - August 2021.

	Physical Restraint and/or Chemical Sedation No. (%) N = 2,977,672		Nested Unadjusted		Nested Adjusted	
	**No** N = 2,930,357 (98.4)	**Yes** N = 47,315 (1.6)	**OR [95% CI]**	**P-Value**	**OR [95% CI]**	**P-value**
**Age**						
18-25	406,601 (13.9)	5,433 (11.5)	0.78 [0.75, 0.82]	<0.001	0.78 [0.75, 0.82]	<0.001
26-35	500,192 (17.1)	9,512 (20.1)	1.02 [0.98, 1.06]	0.39	1.02 [0.98, 1.07]	0.26
36-45	424,836 (14.5)	7,708 (16.3)	Ref	–	Ref	–
46-55	471,800 (16.1)	8,142 (17.2)	0.90 [0.85, 0.94]	<0.001	0.92 [0.88, 0.96]	<0.001
56-64	386,775 (13.2)	5,701 (12.0)	0.84 [0.80, 0.88]	<0.001	0.92 [0.87, 0.96]	<0.001
65+	740,153 (25.3)	10,819 (22.9)	1.07 [1.02, 1.11]	0.001	1.25 [1.20, 1.31]	<0.001
**Sex**						
Female	1,617,824 (55.2)	20,794 (44.0)	0.62 [0.60, 0.63]	<0.001	0.80 [0.78, 0.82]	<0.001
Male	1,312,533 (44.8)	26,521 (56.0)	Ref	–	Ref	–
**Race Ethnicity**						
White Non-Hispanic	1,539,303 (52.5)	25,561 (54.0)	Ref	–	Ref	–
AI/AN Non-Hispanic	8,210 (0.3)	112 (0.2)	0.89 [0.68, 1.17]	0.42	1.04 [0.81, 1.35]	0.74
Asian Non-Hispanic	47,181 (1.6)	360 (0.8)	0.41 [0.36, 0.47]	<0.001	0.58 [0.51, 0.65]	<0.001
Black Non-Hispanic	654,396 (22.3)	11,998 (25.4)	1.04 [1.00, 1.07]	0.023	1.13 [1.10, 1.17]	<0.001
Hispanic or Latina/o/x	599,911 (20.3)	8,168 (17.3)	0.75 [0.72, 0.78]	<0.001	0.90 [0.86, 0.93]	<0.001
Missing or Unknown	13,736 (0.5)	196 (0.7)	1.04 [0.88, 1.23]	0.64	0.97 [0.82, 1.15]	0.74
Native Hawaiian/PI Non-Hispanic	2,844 (0.1)	34 (0.1)	0.63 [0.39, 1.04]	0.07	0.75 [0.47, 1.21]	0.24
Other Non-Hispanic	64,776 (2.2)	883 (1.6)	0.80 [0.73, 0.88]	<0.001	0.96 [0.88, 1.04]	0.31
**Chief Complaint**						
Medical/Non-Behavioral	2,361,820 (80.6)	17,282 (36.5)	0.15 [0.15, 0.16]	<0.001	0.26 [0.25, 0.27]	<0.001
Trauma	441,889 (15.1)	4,910 (10.4)	0.67 [0.64, 0.69]	<0.001	0.52 [0.50, 0.54]	<0.001
Cognitive or Neurologic	196,925 (6.7)	7,483 (15.8)	2.62 [2.55, 2.70]	<0.001	1.73 [1.67, 1.79]	<0.001
Alcohol Drug	110,972 (3.8)	11,426 (24.1)	6.77 [6.56, 6.99]	<0.001	3.67 [3.54, 3.81]	<0.001
Psychiatric	126,300 (4.3)	12,931 (27.3)	7.12 [6.92, 7.32]	<0.001	3.89 [3.76, 4.02]	<0.001
Agitation	7,459 (0.3)	3,811 (8.1)	21.66 [20.44, 22.95]	<0.001	10.87 [10.29, 11.48]	<0.001
**Housing Status**						
Housed	2,878,167 (98.2)	44,155 (93.3)	Ref	–	Ref	–
Unhoused	52,190 (1.8)	3,160 (6.7)	2.90 [2.73, 3.09]	<0.001	1.45 [1.36, 1.54]	<0.001

## Discussion

This study explores the association between patient housing status and ED restraint and sedation use, finding there to be a significant association after adjusting for demographic factors and chief complaints. While a limited number of prior studies have examined the relationship between housing status and restraint use, this study adds an important contribution to this research area for several reasons.

First, the number of visits by unhoused patients included in this study (55,350 visits) is significantly larger than those included in prior studies. For example, Carreras Tartak et al. (2021) and Schnitzer et al. (2020) included 1,838 and 2,003 visits by unhoused patients, respectively, in their analyses [4,6]. Including an unhoused population approximately 27 times larger than those included in prior studies enhances the statistical power and thus, the confidence of detecting a true effect as compared to prior research. This is particularly important given that previous findings on the relationship between housing status and restraint use has been mixed – some studies report a significant association, while others found null results [3–7]. Moreover, since the likelihood of restraint has been found to be associated with several factors disproportionately represented among those who are unhoused (e.g., male sex, substance use, psychiatric conditions, and Black race), this study is valuable in that it helps to deepen and clarify our understanding of the nuanced relationships among these factors.

Second, both restraint/sedation use and unhoused status are independently associated with increased morbidity and mortality [2,16]. Our finding that being unhoused was associated with a higher likelihood of restraint and/or sedation therefore carries important implications, as it highlights a possible mechanism by which health inequities between housed and unhoused individuals may be exacerbated. For example, unhoused individuals experience higher rates of mental health disorders, chronic conditions, and a mortality risk approximately 3.5 times greater than that of housed individuals [17,18]. Given that violent physical restraint is associated with adverse health outcomes, such as physical injury, cardiac arrest, and lasting psychological trauma and distress, our findings underscore how the disproportionate use of restraint/sedation among those who are unhoused may further marginalize and harm an already disadvantaged population.

Lastly, it is important to consider potential long-term implications of these findings and impacts beyond the healthcare setting. Many unhoused patients harbor feelings of distrust and aversion towards the healthcare system due to past mistreatment and stigmatization [10]. Higher rate of restraint/sedation may exacerbate these sentiments, further alienating them from necessary care. Additionally, the use of restraints can extend length of stay in the emergency department, sometimes by more than 24 hours. This presents a particular problem for unhoused individuals. Many are subject to strict shelter curfews and risk losing their place if they do not return on time. If unsheltered, individuals may risk losing belongings at encampments. Consequently, being restrained not only presents physical and psychological risks, but may also complicate individuals’ ability to move from homelessness back to stable housing, potentially prolonging experiences of homelessness.

This study has several limitations. First, it is possible that medications used to evaluate chemical sedation may have been used in a context other than for agitation events, as a direct indicator for agitation does not exist in our health record data. Second, identifying homelessness from the health record is imperfect and likely underestimates homelessness [19]. However, both limitations would result in a bias toward the null and thus the relationship between restraint use and housing status is likely stronger than our model suggests. Third, because our analysis was limited to a single state and regional health care system, our results might not apply to other institutions or geopolitical areas. Fourth, chief complaints were manually grouped based on the listed primary complaint associated with each visit and may not capture nuances of presentations with multiple comorbid issues or potential overlap between medical and behavioral type complaints. Fifth, our results present findings related to use of sedation/restraint but did not capture visits with agitation where restraints/sedation were avoided, potentially due to successful de-escalation. Future work may include investigation of housing status and potential associations with attempts to provide verbal re-direction during agitation episodes. Lastly, while the primary goal in this analysis was to evaluate the association between housing status and restraint/sedation use, interactions between variables such as race, sex, behavioral health diagnoses and housing status undoubtedly exist. Exploring interactions, particularly those related to intersecting vulnerabilities (e.g., race, sex, and housing status) or potential temporal trends over time linked to policy changes or public health crises (e.g., the COVID pandemic), are important areas for future research.

## Conclusion

This study found that unhoused patients were more likely to be restrained and/or sedated in the ED as compared to stably housed patients. While prior research has established associations between demographic factors such as race and ethnicity, age, and gender and restraint use, the influence of social factors, such as housing status, on frequency of restraint use remains underexplored in the literature. This study addresses this gap by analyzing a large and diverse study population to explore this relationship. Additionally, it highlights the need for future research to examine how housing status may interact with or be mediated by additional social and clinical factors.

## Supporting information

S1 Table Descriptive and adjusted multivariable mixed effects logistic regression model of violent physical restraint in the emergency department, January 2013 - August 2021. (DOCX)

S2 Table Descriptive and adjusted multivariable mixed effects logistic regression model of chemical sedation in the emergency department, January 2013 - August 2021. (DOCX)
