# Supplementary material for: Disparities in use of physical restraint and chemical sedation in the emergency department by patient housing status
Source: PLoS One. 2025 Mar 13;20(3):e0319286. doi: 10.1371/journal.pone.0319286 (PMC11906057; doi:10.1371/journal.pone.0319286)
Supplement: S1 Table — (DOCX) [file pone.0319286.s001.docx]

**S1. Table**: Descriptive and Adjusted Multivariable Mixed Effects Logistic Regression Model of Violent Physical Restraint in the Emergency Department, January 2013 - August 2021

|  | **Violent Physical Restraint**  **No. (%)**  N= 2,977,672 | | **Nested Adjusted** | |
| --- | --- | --- | --- | --- |
|  | **No** N=2,961,138 (99.4) | **Yes** N=16,534 (0.6) | **OR [95% CI]** | **P-value** |
| **Age** |  |  |  |  |
| 18-25 | 409,436 (13.8) | 2,598 (15.7) | 0.98 [0.91, 1.05] | 0.59 |
| 26-35 | 505,498 (17.1) | 4,206 (25.4) | 1.10 [1.04, 1.17] | 0.002 |
| 36-45 | 429,301 (14.5) | 3,243 (19.6) | Ref | - |
| 46-55 | 476,964 (16.1) | 2,978 (18.0) | 0.79 [0.74, 0.84] | <0.001 |
| 56-64 | 390,805 (13.2) | 1,671 (10.1) | 0.66 [0.61, 0.71] | <0.001 |
| 65+ | 749,134 (25.3) | 1,838 (11.1) | 0.57 [0.53, 0.61] | <0.001 |
| **Sex** |  |  |  |  |
| Female | 1,633,012 (55.1) | 5,606 (33.9) | 0.59 [0.56, 0.61] | <0.001 |
| Male | 1,328,126 (44.9) | 10,928 (66.1) | Ref | - |
| **Race Ethnicity** |  |  |  |  |
| White Non-Hispanic | 1,556,883 (52.6) | 7,981 (48.3) | Ref | - |
| AI/AN Non-Hispanic | 8,280 (0.3) | 42 (0.3) | 1.04 [0.69, 1.58] | 0.84 |
| Asian Non-Hispanic | 47,434 (1.6) | 107 (0.7) | 0.60 [0.48, 0.75] | <0.001 |
| Black Non-Hispanic | 661,439 (22.3) | 4,955 (30.0) | 1.38 [1.31, 1.45] | <0.001 |
| Hispanic or Latina/o/x | 605,114 (20.4) | 2,968 (18.0) | 0.90 [0.85, 0.96] | 0.001 |
| Missing or Unknown | 13,818 (0.5) | 114 (0.7) | 1.58 [1.27, 1.97] | <0.001 |
| Native Hawaiian/PI Non-Hispanic | 2,864 (0.1) | 14 (0.1) | 0.60 [0.26, 1.39] | 0.23 |
| Other Non-Hispanic | 65,306 (2.2) | 353 (2.1) | 1.16 [1.01, 1.33] | 0.03 |
| **Chief Complaint** |  |  |  |  |
| Medical/Non-Behavioral | 2,376,207 (80.3) | 2,895 (17.5) | 0.11 [0.10, 0.11] | <0.001 |
| Trauma | 445,242 (15.0) | 1,557 (9.4) | 0.39 [0.37, 0.42] | <0.001 |
| Cognitive or Neurologic | 202,073 (6.8) | 2,335 (14.1) | 1.50 [1.41, 1.59] | <0.001 |
| Alcohol Drug | 116,224 (3.9) | 6,174 (37.3) | 3.83 [3.64, 4.04] | <0.001 |
| Psychiatric | 133,555 (4.5) | 5,676 (34.3) | 3.05 [2.89, 3.21] | <0.001 |
| Agitation | 9,319 (0.3) | 1,951 (11.8) | 9.97 [9.32, 10.68] | <0.001 |
| **Homelessness** |  |  |  |  |
| Housed | 2,907,319 (93.2) | 15,003 (90.74) | Ref | - |
| Unhoused | 53,819 (6.8) | 1,531 (9.26) | 1.30 [1.20, 1.41] | <0.001 |
