# Supplementary material for: Disparities in use of physical restraint and chemical sedation in the emergency department by patient housing status
Source: PLoS One. 2025 Mar 13;20(3):e0319286. doi: 10.1371/journal.pone.0319286 (PMC11906057; doi:10.1371/journal.pone.0319286)
Supplement: S2 Table — (DOCX) [file pone.0319286.s002.docx]

**S2. Table**: Descriptive and Adjusted Multivariable Mixed Effects Logistic Regression Model of Chemical Sedation in the Emergency Department, January 2013 - August 2021

|  | **Violent Physical Restraint**  **No. (%)**  N= 2,977,672 | | **Nested Adjusted** | |
| --- | --- | --- | --- | --- |
|  | **No** N=2,936,646 (98.6) | **Yes** N=41,026 (1.4) | **OR [95% CI]** | **P-value** |
| **Age** |  |  |  |  |
| 18-25 | 407,502 (13.9) | 4,532 (11.1) | 0.74 [0.71, 0.78] | <0.001 |
| 26-35 | 501,604 (17.1) | 8,100 (19.7) | 1.02 [0.97, 1.06] | 0.50 |
| 36-45 | 426,000 (14.5) | 6,544 (16.0) | Ref | - |
| 46-55 | 473,009 (16.1) | 6,933 (16.9) | 0.92 [0.88, 0.96] | <0.001 |
| 56-64 | 387,475 (13.2) | 5,001 (12.2) | 0.95 [0.91, 1.00] | 0.06 |
| 65+ | 741,056 (25.2) | 9,916 (24.2) | 1.33 [1.27, 1.39] | <0.001 |
| **Sex** |  |  |  |  |
| Female | 1,619,800 (55.2) | 18,818 (45.9) | 0.85 [0.83, 0.89] | <0.001 |
| Male | 1,316,846 (44.8) | 22,208 (54.1) | Ref | - |
| **Race Ethnicity** |  |  |  |  |
| White Non-Hispanic | 1,542,635 (52.5) | 22,229 (54.2) | Ref | - |
| AI/AN Non-Hispanic | 8,226 (0.3) | 320 (0.2) | 1.08 [0.82, 1.41] | 0.59 |
| Asian Non-Hispanic | 47,221 (1.6) | 10,332 (0.8) | 0.59 [0.52, 0.68] | <0.001 |
| Black Non-Hispanic | 656,062 (22.3) | 7,097 (25.2) | 1.14 [1.10, 1.18] | <0.001 |
| Hispanic or Latina/o/x | 600,985 (20.5) | 159 (17.3) | 0.91 [0.88, 0.95] | <0.001 |
| Missing or Unknown | 13,773 (0.5) | 27 (0.1) | 0.93 [0.77, 1.11] | 0.41 |
| Native Hawaiian/PI Non-Hispanic | 2,851 (0.1) | 766 (1.9) | 0.78 [0.48, 1.28] | 0.33 |
| Other Non-Hispanic | 64,893 (2.2) | 22,229 (54.2) | 0.97 [0.88, 1.06] | 0.50 |
| **Chief Complaint** |  |  |  |  |
| Medical/Non-Behavioral | 2,363,206 (80.5) | 15,896 (38.9) | 0.29 [0.28, 0.30] | <0.001 |
| Trauma | 442,580 (15.1) | 4,219 (10.3) | 0.54 [0.52, 0.56] | <0.001 |
| Cognitive or Neurologic | 197,948 (6.7) | 6,460 (15.8) | 1.74 [1.68, 1.81] | <0.001 |
| Alcohol Drug | 113,537 (3.9) | 8,861 (21.6) | 3.34 [3.21, 3.47] | <0.001 |
| Psychiatric | 127,702 (4.4) | 11,529 (28.1) | 4.43 [4.11, 4.42] | <0.001 |
| Agitation | 7,872 (0.3) | 3,398 (8.3) | 10.62 [10.04, 11.24] | <0.001 |
| **Homelessness** |  |  |  |  |
| Housed | 2,883,969 (98.2) | 38,353 (93.5) | Ref | - |
| Unhoused | 52,677 (1.8) | 2,673 (6.5) | 1.52 [1.42, 1.61] | <0.001 |
